# 10 tips on working with human body donors in medical training and research

**DOI:** 10.1007/s12565-022-00651-0

**Published:** 2022-02-10

**Authors:** Joy Y. Balta, Gabriel Venne, Geoffroy P. J. C. Noël

**Affiliations:** 1 Division of Anatomy, College of Medicine, The Ohio State University, Columbus, OH USA; 2 Department of Anatomy and Cell Biology, Faculty of Medicine and Health Sciences, McGill University, Montreal, QC Canada; 3 Division of Anatomy, School of Medicine, University of California, San Diego, CA USA

**Keywords:** Human body donors, Anatomical research, Medical education, Body donation program

## Abstract

Human body donors selflessly decided to make the ultimate gift to donate their bodies to education. Being on the receiving end, the health sciences education community owes it to the donors to ensure that they are being treated with utmost respect by promoting and developing high ethical standards and maximizing the benefits from this gift. Working with human body donors for research purposes has increased over the years, while regulations associated with these processes did not change. This article draws upon current literature and author’s experiences to offer practical tips for health educators and everyone working with body donors to achieve these goals. We offer 10 practical tips that help in starting the conversation about the best ways to work with body donors to maximize their contribution to health sciences education.

## Introduction

Human anatomical material has been used for many years to learn about the human body (Balta et al. 2015). The sources of these specimens have changed over the years from dubious procurement prior to the nineteenth century to consented body donation. Over the years, human body donors have been strongly associated with the anatomy teaching (Eisma and Wilkinson 2014) and play an important role in clinical training and research (Noël et al. 2021).

Dissecting human bodies has been described as a formative and memorable step in medical training due to the many skills associated with this experience. In addition to the ability to learn anatomy, dissecting a human body improves the learner’s dexterity and also provides an opportunity to introduce topics associated with ethics and professionalism. The importance of working with body donors to integrate basic medical sciences with medical humanities has recently gained more traction and is continually evolving.

Several studies have reported the benefits of working with body donors to enhance clinical training (Yiasemidou et al. 2018; James et al. 2019). Many of these studies reported the use of unembalmed human materials to provide a training environment similar to that of the operating room. While in the past, there was no alternative to unembalmed human materials, recent studies have highlighted the benefits of using soft preservation techniques that allowed for a maximum use of the body donors (Brenner 2014; Balta et al. 2019; Venne et al. 2020; Healy et al. 2015).

Working with human body donors for research purposes has increased over the years; meanwhile, regulations associated with this process have not changed (Noël et al. 2020). At many institutions, research conducted on deceased human bodies is not considered research with human participants and therefore is not reviewed by Internal Review Boards (Bach 2016). This raises many questions and poses challenges to the trust between body donors and the science community (Stimec et al. 2010; Winkelmann 2016).

This article aims to provide all individuals working with body donors a framework that allows them to maximize the benefit from this selfless gift by honoring the donors’ wishes through highest ethical practices.

## Tip 1: consider the medical history of the donors

Human body donors admitted into donation programs, after death, are predominantly with the same age group (Noël et al. 2020). This means that donors will most likely have a complex medical history. Depending on the regulations within each jurisdiction, the level of access to the donors’ medical history could be extremely limited. Donors can disclose part of their medical history when signing the bequeathal forms or at the time of death by the next of kin or health provider. Full disclosure of medical history could compromise the donor’s anonymity as outlined in some bequeathal forms (Jones and King 2017).

When working with body donors for educational purposes, the donors’ medical history could provide a unique learning opportunity. When teaching anatomy to health sciences students, the donors’ medical conditions will provide faculty with an opportunity to integrate clinical correlates to the anatomy learning process (Collins et al. 2018). This has proven to be an advantageous teaching approach as it reminds the students about the reason why it is important to learn human anatomy as part of their career.

Not having access to the donors’ medical history might pose some challenges. Some of these challenges are associated with the embalming process. Based on the authors’ experience, unknown pathologies in a donor might alter the outcomes of the embalming process and therefore affecting the period under which the anatomical material can be used. Moreover, the donors’ unknown medical history might increase the limitations of a research study as these pathologies might have an impact on the findings of the study. Therefore, it is important to gather as much information as possible on the donor’s medical background.

## Tip 2: embrace variations in the human body

One of the advantages of working with human body donors is the uniqueness of every individual. Unlike animals, the genotype and phenotype of the body donors are not homogenous. That means that the shape and size of different structures within the body could be different, and sometimes, there could be small differences in the anatomy. This is usually referred to as anatomical variations which are really important for student learning and needs to be further highlighted. Working with body donors and medical imaging is the only way to demonstrate variations as this would not be possible with plastic models and most technology-based learning.

In a time when medical care is moving toward personalized medicine, it is crucial to benefit from these anatomical variations to help train the next generation of clinicians. Recent studies have highlighted the benefits of incorporating human material in the residency training and other graduate medical education programs (Alraddadi 2021). Moreover, this can also have a huge role in the testing and development of new medical equipment as these variations are extremely helpful for education, training, and research. It is important to be aware of some of the limitations that anatomical variations may introduce to different research studies. This could primarily impact the validation and reproducibility of research studies.

## Tip 3: plan on collaborating

Working with anatomical material offers invaluable opportunities for education and research but also comes with limitations. One example is accessibility, as the demand is always higher than the available material. Several factors impact the decision to donate a human body such as personal beliefs, religion, finances, or the level of awareness. Therefore, access to body donors could differ significantly between jurisdictions (i.e., countries, state, provinces, or institutions) (Habicht et al. 2018). For this reason, collaboration is essential between body donation programs and anyone desiring to work with human tissue.

When working with donors for educational purposes, the availability of donors at any given time should be considered. It is important to establish those collaborations at the early stages of project development to determine the possibilities and refine the project accordingly. Many variables need to be considered and monitored by the body donation program team that could be crucial for research and training such as the availability, quality, and state of the tissue on top of all other legal paperwork.

Several challenges can be identified when working with body donors for research purposes. One of these challenges is the sample size that has an impact on the study design. While working with animal subjects, sample size can often be changed according to the needs with any needed specifications. This is not the case when working with human body donor samples. It is important for researchers to factor in the time needed to recruit samples for this type of research. Having a well-designed and flexible plan is crucial for the success of the research project that could be implemented as soon as the samples become available.

In addition to the collaboration between body donation program and end users, it is important to be aware of further collaboration with other end users of human materials. This will be needed when trying to maximize the benefits of this generous donation by allowing multiple end users to work with one donor. As being the entity entrusted with this donation and responsible for overseeing the use of the anatomical material, this will require coordinating between different groups to successfully achieve this goal.

## Tip 4: invest in developing preservation techniques

With the constantly increasing demands of anatomical material (Noël et al. 2020), institutions have to be creative in defining ways to optimize the benefits from this donation. On that front, tremendous advancement in the preservation method has been made during the last decade where fresh and formaldehyde-embalmed tissues are not the only available options anymore. A variety of embalming techniques offering a large spectrum of tissue quality is now available (Balta et al. 2015; Brenner 2014; Crosado et al. 2020) from soft-embalmed which is providing a more realistic experience for advanced medical training sessions, to formaldehyde-embalmed that has been historically kept for undergraduate curriculums. Since the tools are available, there is a need to optimize the way under which donation programs operate.

Several studies have reported their institutional experience in using alternative preservation techniques (Eisma et al. 2013; Sanders and Bazira 2018). This demonstrates a successful transition from formaldehyde-embalmed to soft-embalming specimens for undergraduate medical training. This change in the embalming approach creates an opportunity to develop a hierarchy of users, which would need to be validated, where minimally invasive training sessions and/or research projects could be performed first on the donor, followed by more invasive activities such as full-body dissection (Balta et al. 2015). As a result, several cohorts of learners could benefit from this unique training experience from the same donor.

Institution, curriculum designers and researchers should work collaboratively in defining the best sequence possible. With more understanding of how different embalming techniques interfere with one another, a suggested tissue usage sequence could be optimized. This would start with none-invasive research that could be performed on fresh state tissue, then advanced minimally invasive training could be subsequently performed on soft-embalmed tissue, and then the donor would be formaldehyde-embalmed for undergraduate medical education.

## Tip 5: publish all new evidence

While body donors have played a significant role in the scholarship of teaching in anatomy education, several gaps could be identified in their contributions to the scholarship of discovery (Benninger 2013). The use of anatomical material has often been entrenched out of sight of the population where most of the technical work is not disseminated among the community of practitioners. Researchers interested in using such material should be sensitized to the reality that body donors are willingly bequeathing their bodies to not only educate but also help in the scientific quest for better treatment.

Anatomical research on how to use body donors is now more important than ever. Medical education and public health journals are welcoming such research. It is a time of transparency. New evidences on improved preservation techniques, ways to present body donors with the self-expression of the persons in mind, or even, multi-cultural lenses on the practices need to be shared (Amath et al. 2017). Scholarship in anatomical sciences is being recognized with the emergence of new anatomical journals with high impact factors, reflecting this new reality. Anatomy touches upon many fields of society; from public health to psychology. The way we interact with our dead has always been a great indicator of the values of our societies.

## Tip 6: honor and acknowledge the donors

Human body donors make the ultimate and final gift by donating their bodies for education and research and everyone working with body donors should do their best to honor. One of the ways to do so is by being good stewards of this donation by maximizing the contributions of these donors to education and research. This requires putting plans in place to allow multiple users to benefit from this donation and extend the period of use and to ensure that the end user is acknowledging this generous act by communicating the wishes of the donors to contribute to education and research (Iwanaga et al. 2021).

This gift puts the primary stewardship responsibility on the leadership within body donation programs to develop such plans. Some of these plans have been discussed in the literature where non-invasive approaches can be performed on the donor followed by minimal invasive procedures, and finally, full-body dissection can take place. While researchers might consider the option of reusing the donors in different projects, technical staff need to follow the maintenance protocol to prevent decomposition of the human body. However, maximizing the contributions of donors to education and research should not align with any financial gain. Isolating tissue to allow educators and researchers to utilize different body regions of a donor is a good example of this stewardship, meanwhile increasing profit and financial gain should not motivate this practice. Ensuring that donors’ contribution is ethically maximized will not only honor the donors, it will also help alleviate some of the tension caused by working with deceased human bodies instead of relying on detachment and the objectification of the human body. It is important to acknowledge the body donors and their families in any scholarship such as research outputs. Moreover, images or videos captured should be protected and acknowledged the sources of the material in ways similar to patients’ electronic health records.

Another way to acknowledge the generosity of donors is by communicating their contributions to their families and loved ones (Winkelman et al. 2016). For this reason, it is important to organize a memorial service where students, researchers, educators, and clinicians can come together to publicly express their gratitude and celebrate the contributions of the body donors (Ghosh 2017; Rocha et al. 2020).

## Tip 7: engage in ethical practice

While human materials donated for organ or tissue transplantation is highly regulated and strictly enforced, this is not the case for full human body donation. The lack of legal oversight in some countries leads to limited policies and procedures within institutions (Habicht et al. 2018). Moreover, the legal ramification associated with whole-body donation is mainly restricted to the donation and disposition of the remains, while ethical practice needs to be considered when working with body donors (Jones and Whitaker 2012). With increasing demands of anatomical material, this gray zone around the work with body donors allows for-profit anatomical material companies to run business (Champney 2016; Champney et al. 2019). Serious questions could be raised on how ethical the procurement and the use of human material is conducted within for-profit companies. Considering the extent by which body donors are being sent worldwide to meet the demand over the globe and the moral and ethical implications of such trade (Champney et al. 2019), it is important that requests for human material be placed or redirected to local body donation programs. Despite the broad regulation spectrum across countries and jurisdictions, moral and ethical considerations should always prevail (Jones 2016). Moreover, resources and support should be invested in helping countries and jurisdictions in need to build their own body donation programs instead of relying on international programs promoting commercialization of bodies. Arguments could also be made to advocate for subsidization of the body donation programs to prevent those to focus on generating revenues for cost recovery and expansion of their activities.

When making this, generous gift donors are entrusting their bodies to the donation program. For this reason, the highest ethical standards must be developed and practiced when working with body donors (Bach 2016). One way of achieving this goal is by developing an oversight committee that is able to oversee the operations of the body donation program. This committee can include representative from different backgrounds such as law, ethics, research, and even individuals that have donated their bodies to education and research. When it comes to research, deceased human bodies are not considered as human subject by many Internal Review Boards (IRB), and therefore, research with deceased human bodies is not reviewed for ethical purposes. This gap could also be covered by the work of the oversight committee that can play a role in reviewing and providing ethical approval for research conducted on body donors.

Body donation programs can lead and promote best ethical practices, but to be successful in achieving this goal, all individuals working with body donors must self-monitor the use of the donor’s tissue ethically and in a manner that embraces human dignity.

## Tip 8: walk the line between empathy and clinical detachment

Working with deceased human bodies has been primary known for improving the knowledge about the anatomy of the human body and therefore focusing on the cognitive and psychomotor domains of learning. More recently, working with body donors has become a natural place to develop and foster health care professionals who are technically competent, as well as emotionally equipped, to provide patients with compassionate care. The setting allows development of emotional skills from the affective domain such as empathy and clinical detachment (Hildebrandt 2010), and reflection on death (Patel and Moxham 2008; Arraez-Aybar et al. 2008). In a time when death is often abstracted or even targeted, sensitization of trainees to a passing individual and how to work around their own emotion while practicing surgical approaches can be an added value compared to other simulation tools.

Another underutilized advantage of working with body donors is the ability to teach ethics and professionalism in an integrated approach as part of the medical curriculum. Using anatomical material can offer trainees the opportunity to revisit important concept of consent, respect, moral obligation, ethical ramifications, and disparity in health. Humanities can be explored during sessions with body donors to augment the benefit of just using material for material’s sake. Ultimately, this will instill another level of professional behavior in any of the endeavor related to human bodies.

## Tip 9: engage the community

An outstanding educator is a life-changing individual that empowers students to become better citizens, raising the next generation. Donors are those educators that silently, with their ultimate donation, contribute to the advancement of their community and society. While the donation is being made to the university, the university should play the role of the executor of the donors’ wishes, rather than being the sole beneficiary of this gift. For this reason, benefiting from this gift should not be limited to those who can afford having access to this resource, but should also expanded to the community at large who should also benefit from it.

Body donation programs should welcome and encourage non-profit outreach activities to share this unique opportunity with the community and society. An example would be providing high school students with an opportunity to learn from a body donor or other non-profit organization providing opportunities of learning through anatomical material in activities carefully designed in relevance to their level and field of practice. Educational resources developed through this gift should also be accessible to those who cannot afford to benefit from this donation. This will increase the number of beneficiaries and extends the impact that this donor can have on society, thus respecting their wishes (Meyer et al. 2018).

## Tip 10: growth and development

Human body donors have been used for centuries as a tool to learn about the human body. While they remain as a superior tool to advance education and training, the way we integrate their use in more modern activities can be improved. As advancements in technology provide new learning and training tools, the way we approach the utility of human bodies needs to meet the modern-day demands. Many institutions struggle to maintain their body donation programs, as some might view the use of body donors as an outdated tool that is expiring and becoming redundant. The science community shares some of the responsibility of these views as little to no research has been dedicated to improving and revolutionize the gift that is left by body donors (Jones 2016). As a community, we owe it to our donors to dedicate resources that will help bridge the gap between the traditional ways of working with body donors and the modern-day needs as discussed in the tips above. This will help in the way we prepare and become better stewards of this generous donation.

Another area of growth that needs to be addressed is access to information about donation which will provide people from diverse backgrounds with access to donation*.* We need to communicate with people from all socioeconomic status and underrepresented minorities who may have received biased and unequal access to medical treatment resulting in lower life expectancy (Campbell et al. 2020; da Rocha et al. 2017; Rajah et al. 2014; Steinecke and Terrell 2010). Studies have shown that most (Boulware et al. 2004; Wadhwani et al. 2021) of the human body or organ donors or people willing to donate their bodies or organs in the US are non-Hispanic White. This is due to the systemic racism embedded in our society and dedicated resources are needed to break this perpetual cycle. This disparity in body donation also impacts the anatomy learning space. Body donation diversity may provide more of an opportunity for medical students to unlearn bias-driven untruths such as Black people have thicker skin and have a higher tolerance for pain than White people. While the gross anatomy does not differ between individuals from different races, yet representation does matter for everyone involved.

## Conclusion

Everybody agrees that human body donors are making the ultimate and selfless gift by contributing to education and research by offering their own bodies. It is our responsibility to do all what we can to honor their wishes and ensure that they are treated respectfully and with dignity. These 10 tips come to support the effort toward increasing awareness on the need for an open dialog between everyone involved to ensure the donors’ best interest.
